# Correlation of Myocardial Strain and Late Gadolinium Enhancement by Cardiac Magnetic Resonance After a First Anterior ST-Segment Elevation Myocardial Infarction

**DOI:** 10.3389/fcvm.2021.705487

**Published:** 2021-07-02

**Authors:** Shiqin Yu, Jinying Zhou, Kai Yang, Xiuyu Chen, Yucong Zheng, Kankan Zhao, Jialin Song, Keshan Ji, Peng Zhou, Hongbing Yan, Shihua Zhao

**Affiliations:** ^1^State Key Laboratory of Cardiovascular Disease, MR Center, Fuwai Hospital, National Center for Cardiovascular Diseases, Chinese Academy of Medical Sciences and Peking Union Medical College, Beijing, China; ^2^Stata Key Laboratory of Cardiovascular Disease, Coronary Heart Disease Center, Fuwai Hospital, National Center for Cardiovascular Diseases, Chinese Academy of Medical Sciences and Peking Union Medical College, Beijing, China; ^3^Paul C. Lauterbur Research Center for Biomedical Imaging, Shenzhen Institutes of Advanced Technology, Chinese Academy of Sciences, SZ University Town, Shenzhen, China; ^4^Fuwai Hospital, Chinese Academy of Medical Sciences, Shenzhen, China

**Keywords:** ST-segment elevation myocardial infarction, magnetic resonance imaging, strain, late gadolinium enhancement, left ventricle

## Abstract

**Objectives:** To investigate the correlation of cardiac magnetic resonance (CMR) feature-tracking with conventional CMR parameters in patients with a first anterior ST-segment elevation myocardial infarction (STEMI).

**Methods:** This sub-analysis of OCTAMI (Optical Coherence Tomography Examination in Acute Myocardial Infarction) registry included 129 patients who finished a CMR examination 1 month after a first anterior STEMI. Cine images were applied to calculate both global and segmental left ventricular peak strain parameters. The patients were divided into two groups by left ventricular ejection fraction (LVEF) and compared with 42 healthy controls. Segmental late gadolinium enhancement (LGE) was graded according to LGE transmurality as follows: (1) >0 to ≤ 25%; (2) >25 to ≤ 50%; (3) >50 to ≤ 75%; (4) >75%. Left ventricle was divided into infarcted, adjacent, and remote regions to assess regional function.

**Results:** Compared with controls, global radial (28.39 ± 5.08% vs. 38.54 ± 9.27%, *p* < 0.05), circumferential (−16.91 ± 2.11% vs. −20.77 ± 2.78%, *p* < 0.05), and longitudinal (−13.06 ± 2.15 vs. −15.52 ± 2.69, *p* < 0.05) strains were impaired in STEMI patients with normal LVEF (≥55%). Strain parameters were strongly associated with LGE (radial: *r* = 0.65; circumferential: *r* = 0.69; longitudinal: *r* = 0.61; all *p* < 0.05). A significant and stepwise impairment of global strains was observed in groups divided by LGE tertiles. Furthermore, segmental strain was different in various degrees of LGE transmurality especially for radial and circumferential strain. Strains of adjacent region were better than infarcted region in radial and circumferential directions and worse than remote region in all three directions.

**Conclusion:** Global and regional strain could stratify different extent and transmurality of LGE, respectively. Although without LGE, adjacent region had impaired strains comparing with remote region.

## Introduction

Cardiac magnetic resonance (CMR) imaging is considered a gold standard for quantification of cardiac function integrating myocardial tissue characterization (1). Left ventricular ejection fraction (LVEF) assessment is indicated for risk stratification and prognostic management in ST-segment elevation myocardial infarction (STEMI) patients (2). However, LVEF is neither able to detect regional variations in myocardial contractility nor identify subtle but important contractile abnormalities (3, 4). Moreover, patients with heart failure can present a preserved LVEF. Late gadolinium enhancement (LGE) provides delineation of infarcted myocardium *in vivo* of which myocardial contractility is unlikely restored after coronary revascularization (5).

Moreover, myocardial deformation can be evaluated by CMR. Recently, strain analysis by CMR feature-tracking, using routinely acquired cine images, has been increasingly conducted to detect subtle and regional myocardial dysfunction in a variety of cardiovascular diseases including myocardial infarction (6, 7). Unlike LVEF which reflects contractile function by volumetric changes, strain is a more in-depth evaluation studying three different directions of myocardial deformation corresponding to geometry of myocardial fibers. In this regard, CMR feature-tracking is a potential supplement to LVEF and LGE for assessment in STEMI patients.

Therefore, we aimed to investigate whether strain analysis by CMR feature-tracking could provide complementary evaluation value in STEMI patients on top of conventional CMR parameters including LVEF and LGE. We hypothesized that myocardial strain assessed by CMR feature-tracking could be more sensitive to detect early decline in left ventricular (LV) function with preserved LVEF, quantify regional dysfunction, and discriminate different degrees of myocardial infarction assessed by LGE.

## Methods

### Study Population

The present retrospective study was a sub-analysis from OCTAMI (Optical Coherence Tomography Examination in Acute Myocardial Infarction) registry (clinical trial unique identifier: NCT03593928), which continuously enrolled a prospective cohort of STEMI patients for evaluating culprit lesions by optical coherence tomography. The major inclusion criteria for OCTAMI were as follows: (1) age ≥18 years; (2) presented with persistent chest pain lasting more than 30 min with ST-segment elevation >0.1 mV in at least two contiguous leads or new left bundle-branch block on the 18-lead electrocardiogram and elevated troponin I level (2); (3) referred to primary percutaneous coronary intervention. Patients were qualified for the current study if they (1) presented with a first STEMI due to left anterior descending coronary artery and (2) finished a CMR examination at 1 month after index procedure. Forty-two age- and sex-matched healthy subjects were recruited as controls. This study was approved by the review board of the local hospital and all participants provided written informed consent.

### CMR Imaging

CMR imaging was performed on a 3.0-Tesla scanner (Discovery MR750; GE Healthcare, Milwaukee, USA) with a phased-array cardiovascular coil, using electrocardiographic and respiratory gating. The protocol mainly consisted of cine imaging and LGE imaging for analysis. Cine images were acquired in three long-axis views (LV two-chamber, three-chamber, and four-chamber) and short-axis views encompassing the entire LV using balanced steady-state free precession sequence (b-SSFP). Typical imaging parameters were field of view = 320 × 320 mm, matrix = 224 × 192, repetition time (TR) = 3.3 ms, echo time (TE) = 1.7 ms, flip angle = 50°, temporal resolution = 46–60 ms, slice thickness = 8 mm, and slice gap = 2 mm. LGE images were acquired 10–15 min after intravenous administration of gadolinium-DTPA (Magnevist; Bayer, Berlin, Germany) at a dose of 0.2 mmol/kg, using a segmented phase-sensitive inversion recovery sequence at the same views as cine images in end diastole. Typical imaging parameters were field of view = 360 × 360 mm, matrix = 224 × 192, TR = 6.0 ms, TE = 2.8 ms, flip angle = 25°, slice thickness = 8 mm, slice gap = 2 mm, and TI = 300 ms.

### CMR Analysis

All the analyses were conducted using commercial software CVI42 (Circle Cardiovascular Imaging, Calgary, Canada) by investigators with more than 3 years' experience. Endocardial and epicardial contours of LV myocardium were manually traced on short-axis cine at end diastole and end systole, respectively, and cardiac functional parameters were computed automatically. Papillary muscles were assigned to the LV volume. For quantification of contrast enhancement, outline of left ventricular myocardium was manually depicted and LGE was detected by +5 SDs over the signal intensity of normal myocardium (8, 9). LGE results were recorded as percentage of enhanced myocardial volume of left ventricle. Feature-tracking performed on three long-axis cines and short-axis cine to calculate LV peak strain parameters, including global radial strain (GRS), global circumferential strain (GCS), and global longitudinal strain (GLS). All endocardial and epicardial borders of LV throughout the cardiac cycle were automatically tracked by contours manually delineated at end diastole. After that, all the boundary points were checked and the contours would be adjusted if necessary. American Heart Association (AHA) 16-segment model was used to generate segmental results (10). Segments were graded according to LGE transmurality in end-diastole as follows: (1) >0 to ≤ 25%, (2) >25 to ≤ 50%, (3) >50 to ≤ 75%, and (4) >75%. Because the culprit lesion was left anterior descending coronary artery in present patients, we divided LV into three regions by combining the method described by Götte et al. (11) and AHA 16-segment model: infarcted region (segments 1, 2, 7, 8, 13, 14)—LGE distributed region, adjacent region (segments 3, 6, 9, 12, 15, 16)—contiguously to the infarcted region, and remote region (segments 4, 5, 10, 11)-−180° opposite from the infarct. Two radiologists with 3- and 5-year experience of CMR imaging assessed strain parameters in 15 random patients independently for inter-observer analysis. 3 months later, one of the investigators repeated assessment to determine the intra-observer variability.

### Statistical Analyses

Continuous variables were expressed as mean ± SD or median values with interquartile range (IQR) depending on normality variables. Correspondingly, *t*-test or Mann–Whitney *U* test was applied to compare two groups; one-way ANOVA with *post hoc* LSD tests or Kruskal–Wallis H test was performed for comparisons of three groups. Categorical variables were reported as exact numbers with percentages and χ^2^ test or Fisher exact test was conducted for comparison. Linear regression analyses were performed to determine the association between strain parameters and LGE. Receiver operating characteristics analysis was used to define the optimal cut-off values by the Youden Index and to quantify discriminative power. Inter- and intra-observer analyses were conducted by intraclass correlation coefficient. Statistical analyses were performed using IBM SPSS Statistics 23.0 (Armonk, NY) and MedCalc 16.8.4 (Ostend, Belgium). A two-tailed *p*-value <0.05 was considered statistically significant.

## Results

### Baseline Characteristics

**Figure 1:**
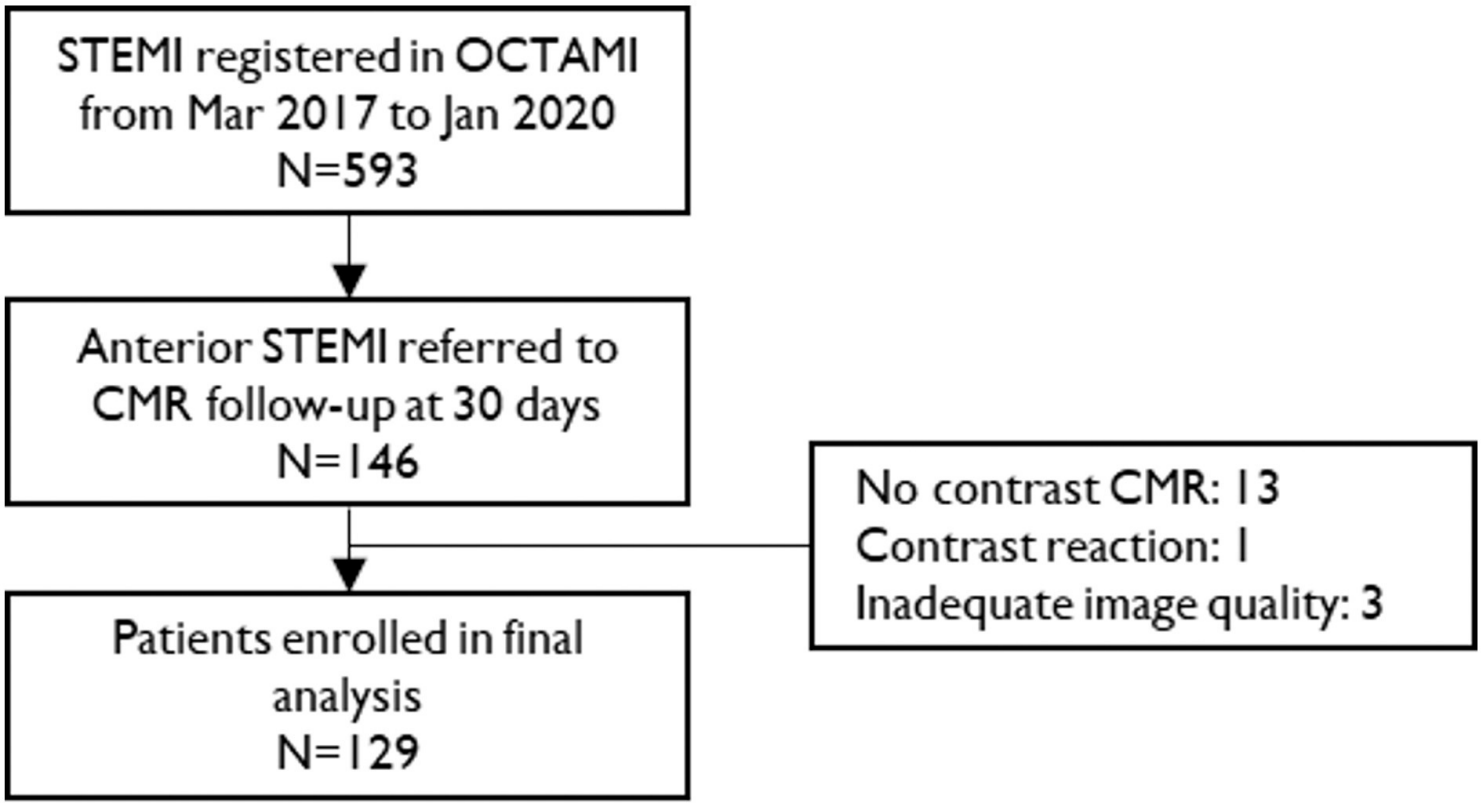
Study flow chart. STEMI, ST-segment elevation myocardial infarction; CMR, cardiac magnetic resonance.

A total of 129 STEMI patients (age 55 years, IQR, 48–63 years; 112 men) and 42 healthy controls (age 53 years, IQR, 48–60 years; 37 men) were included in the study (Figure 1). Table 1 summarizes the baseline characteristics of patients' population. Patients had a high prevalence of hyperlipidemia (72%) and 87% of the patients were male. All participants presented with a first anterior STEMI due to left anterior descending artery. However, 47 patients (36%) had two diseased vessels and 33 (26%) had three diseased vessels.

**Table 1:** Baseline characteristics of the study population.

	**Total STEMI (*n* = 129)**	**LVEF <55% (*n* = 85)**	**LVEF ≥55% (*n* = 44)**	***P-*value**
Age, years	55 (48–63)	52 (46–63)	56 (50–62)	0.26
Male, *n* (%)	112 (87)	71 (84)	41 (93)	0.12
Body mass index, kg/m^2^	25.4 (23.8–27.7)	25.5 (23.8–27.9)	25.4 (23.2–27.1)	0.52
Hypertension, *n* (%)	64 (50)	44 (52)	20 (46)	0.50
Hyperlipidemia, *n* (%)	93 (72)	61 (72)	32 (73)	0.91
Diabetes mellitus, *n* (%)	40 (31)	25 (29)	15 (34)	0.59
Smoker, *n* (%)	92 (71)	57 (67)	35 (80)	0.14
Ischemia time, h	5.0 (3.0–7.8)	5.0 (3.0–7.3)	5.0 (3.0–8.0)	0.81
TIMI flow pre-PCI, *n* (%)				0.02
0	71 (55)	54 (63)	17 (39)	
1	10 (8)	6 (7)	4 (9)	
2	15 (12)	10 (12)	5 (11)	
3	33 (25)	15 (18)	18 (41)	
TIMI flow post-PCI, *n* (%)				1.00
0	2 (1)	1 (1)	1 (2)	
1	0 (0)	0 (0)	0 (0)	
2	1 (1)	1 (1)	0 (0)	
3	126 (98)	83 (98)	43 (98)	
Number of diseased vessels				0.63
1	49 (38)	32 (38)	17 (39)	
2	47 (36)	31 (36)	16 (36)	
3	33 (26)	22 (26)	11 (25)	
Admission creatinine, μmol/L	74.6 (65.2–85.2)	74.8 (65.0–86.0)	73.1 (66.2–84.4)	0.88
Peak cTnI, ng/mL	27.0 (9.5–53.3)	33.5 (18.2–60.1)	13.5 (6.6–31.6)	0.001
Time of peak cTnI, h	20 (14–27)	21 (15–27)	19 (12–27)	0.38
Peak NT-proBNP, pg/mL	1,364.5 (729.1–2,889.1)	1,666.1 (915.9–3,215.3)	914.7 (404.7–2,072.2)	0.001
Time of peak NT-proBNP, h	29 (22–42)	32 (24–49)	26 (19–32)	0.004

*PCI, percutaneous coronary intervention; cTnI, cardiac troponin I; NT-proBNP, N terminal pro B type natriuretic peptide*.

### CMR Parameters

CMR examinations were conducted 33 (IQR, 30–38) days after index events. The STEMI patients were divided into two groups by LVEF (55%) and compared with healthy subjects. An overview of assessed CMR parameters is presented in Table 2. LGE distribution was consistent with left anterior descending coronary territory. Compared with controls, all the CMR parameters were impaired in the whole STEMI group (all *p* < 0.001). Conventional cardiac function parameters (except cardiac output) were similar between patients with LVEF ≥55% and controls (all *p* > 0.05), but GRS, GCS, and GLS were impaired in patients with LVEF ≥55% (Supplementary Figure 1, all *p* < 0.001). Furthermore, all strain parameters of STEMI patients with LVEF <55% were much worse than LVEF ≥55% group (Supplementary Figure 1, all *p* < 0.001). It is worth noting that the percentage of LGE in LVEF <55% group was significantly more than LVEF ≥55% group (*p* < 0.001). Therefore, the correlation of myocardial strain and extent of LGE was investigated further.

**Table 2:** CMR parameters of the study population.

	**STEMI**			**Controls (*n* = 42)**
	**Total (129)**	**LVEF <55% (*n* = 85)**	**LVEF ≥55% (*n* = 44)**	
LVEF, %	51 (42–57)^*^	45 (38–51)^†^	60 (56–63)^‡^	63 (59–67)
SV, mL	72.58 ± 17.48^*^	68.71 ± 16.56^†^	80.05 ± 16.96^‡^	83.66 ± 15.03
EDV, mL	150.54 ± 34.13^*^	159.10 ± 34.25^†^	134.02 ± 27.41^‡^	132.50 ± 23.62
ESV, mL	71.9 (57.5–96.3)^*^	88.4 (71.2–107.0)^†^	54.8 (43.6–63.9)^‡^	47.4 (41.1–57.3)
CO, L/min	4.70 ± 1.19^*^	4.57 ± 1.25^†^	4.96 ± 1.00^†^	6.02 ± 1.09
LGE, %	12.04 (5.80–19.82)	17.14 (9.67–24.14)	6.38 (1.88–11.60)^‡^	–
GRS, %	22.39 ± 6.60^*^	19.28 ± 4.95^†^	28.39 ± 5.08^†‡^	38.54 ± 9.27
GCS, %	−14.11 ± 3.19^*^	−12.66 ± 2.64^†^	−16.91 ± 2.11^†‡^	−20.77 ± 2.78
GLS, %	−11.46 ± 2.64^*^	−10.64 ± 2.50^†^	−13.06 ± 2.15^†‡^	−15.52 ± 2.69

*CMR, cardiac magnetic resonance; STEMI, ST-segment elevation myocardial infarction; LVEF, left ventricular ejection fraction; SV, stroke volume; EDV, end-diastolic volume; ESV, end-systolic volume; CO, cardiac output; LGE, late gadolinium enhancement; GRS, global radial strain; GCS, global circumferential strain; GLS, global longitudinal strain*.

* *p < 0.05 when compared with the controls between 2 groups (the total STEMI group and the controls)*.

† *p < 0.05 when compared with the controls among three groups (the STEMI LVEF <55% group, the STEMI LVEF ≥55% group, and the controls)*.

‡ *p < 0.05 when compared with the STEMI (LVEF <55%) group among three groups (the STEMI LVEF <55% group, the STEMI LVEF ≥55% group, and the controls)*.

### Association Between LV Strain and LGE

**Figure 2:**
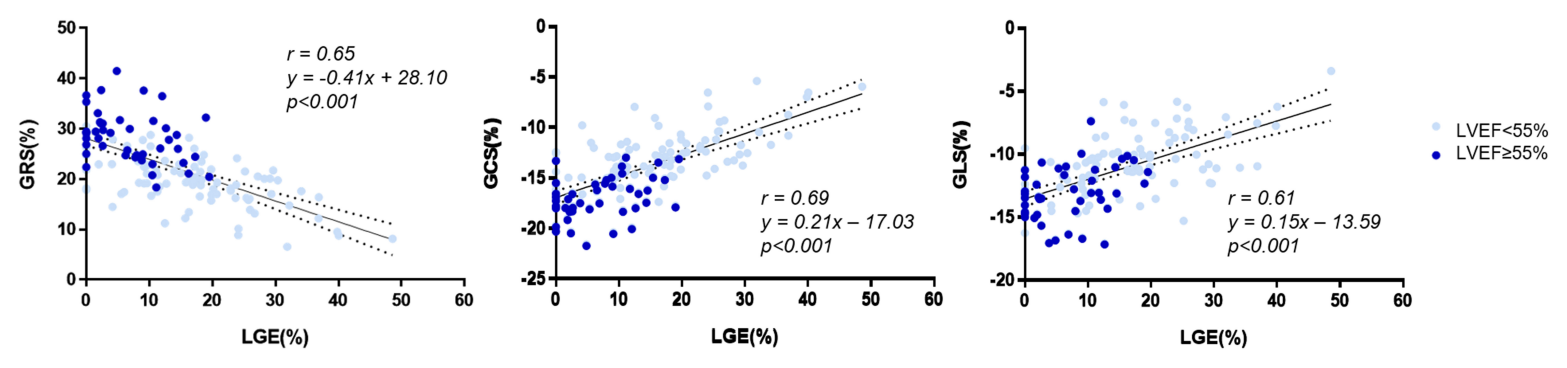
Correlation of LGE and global strain. LGE, late gadolinium enhancement; GRS, global radial strain; GCS, global circumferential strain; GLS, global longitudinal strain.

**Figure 3:**
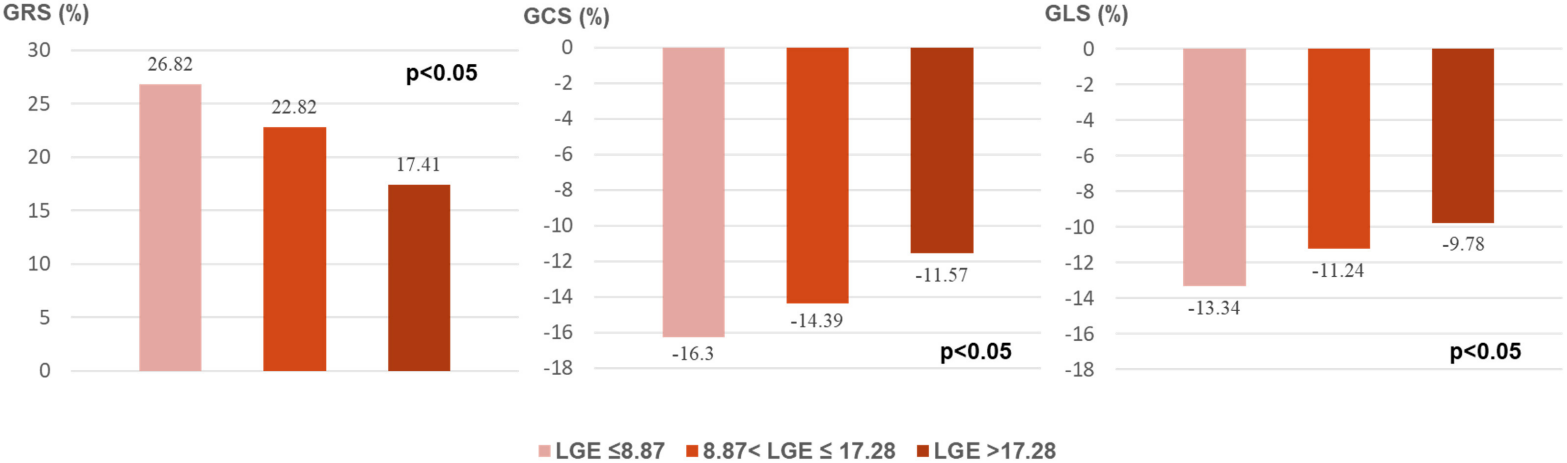
Global strains in LGE tertiles. LGE, late gadolinium enhancement; GRS, global radial strain; GCS, global circumferential strain; GLS, global longitudinal strain.

As Figure 2 shows, all global strain parameters were strongly associated with LGE extent (GRS: *r* = 0.65, β = −0.41, *p* < 0.001; GCS: *r* = 0.69, β = 0.21, *p* < 0.001; GLS: *r* = 0.61, β = 0.15, *p* < 0.001). When dividing the patients by LGE tertiles, the increase in LGE extent was correlated to a significant and stepwise impairment of global strains (Figure 3): the average strain values for LGE tertiles were 26.82, 22.82, and 17.41% for GRS; −16.3, −14.39, and −11.57% for GCS; and −13.34, −11.24, and −9.78% for GLS.

**Figure 4:**
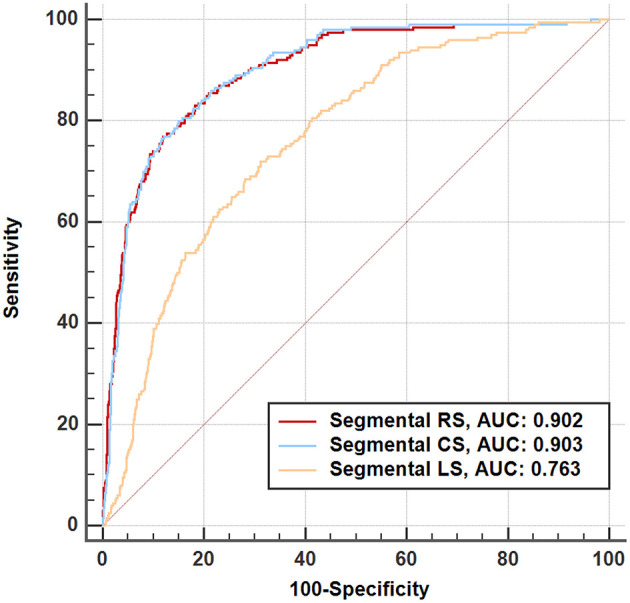
Receiver operating characteristic analysis for discriminating segmental late gadolinium enhancement >50%. RS, radial strain; CS, circumferential strain; LS, longitudinal strain.

**Figure 5:**
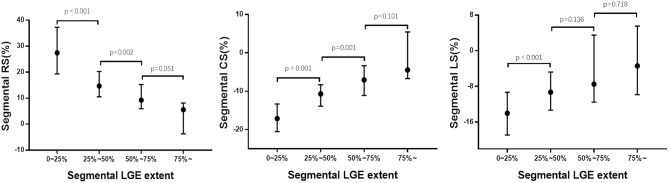
Association between segmental strain and LGE extent. RS, radial strain; CS, circumferential strain; LS, longitudinal strain; LGE, late gadolinium enhancement.

For segmental results, the receiver operating characteristic curve analysis demonstrated that all segmental strain parameters were good discriminators for segmental LGE >50% (Figure 4), and radial strain [cut-off value: 12.89%, sensitivity: 77%, specificity: 88%, area under curve (AUC): 0.902] and circumferential strain (cut off value: −10.20%, sensitivity: 80%, specificity: 85%, AUC: 0.903) performed better than longitudinal strain (cut-off value: −9.98%, sensitivity: 72%, specificity: 69%, AUC: 0.763). Furthermore, Figure 5 illustrates that segmental strain was different in various degrees of LGE transmurality especially for radial and circumferential strain. The more transmural the segmental LGE, the worse the segmental strain.

### Strain Parameters in Different Regions

**Figure 6:**
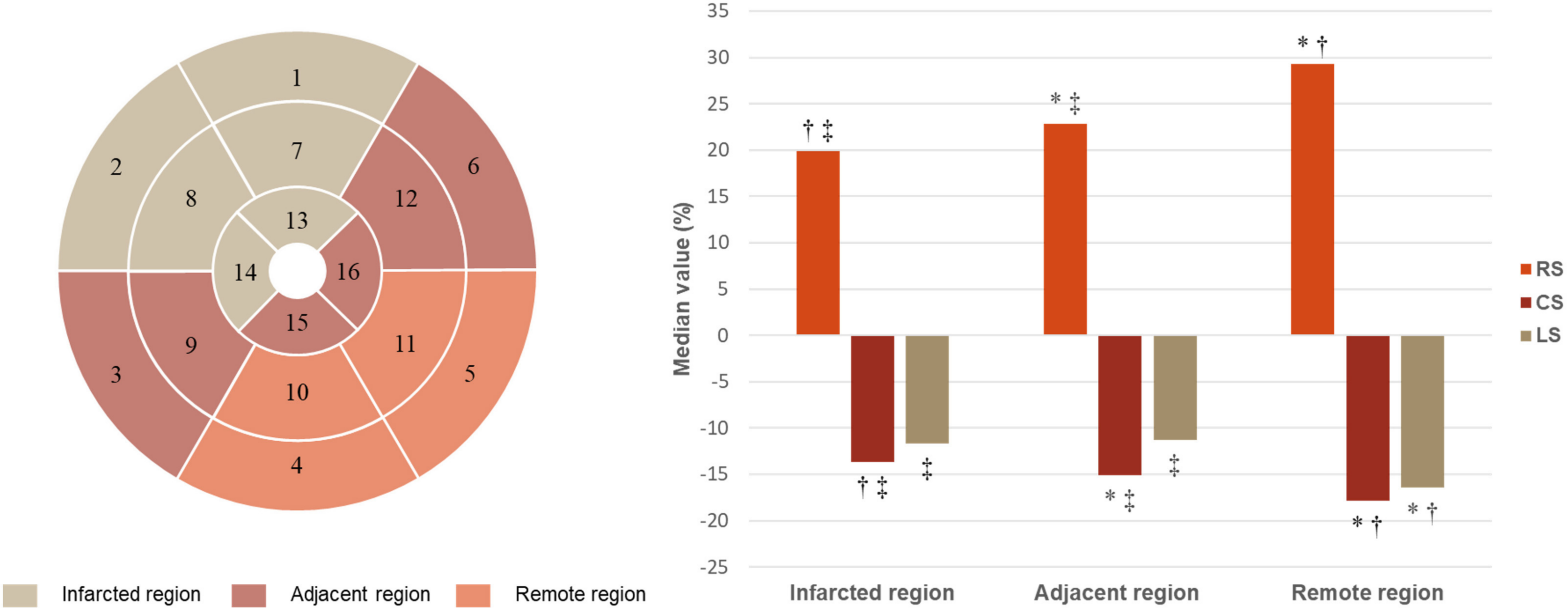
Strain in infarcted, adjacent, and remote regions. RS, radial strain; CS, circumferential strain; LS, longitudinal strain. *p < 0.05 vs. infarcted region; ^†^*p* < 0.05 vs. adjacent region; ^‡^*p* < 0.05 vs. remote region.

The strain of remote region, adjacent region, and infarcted region presented a significant and stepwise impairment in radial (median value: 29.30, 22.84, and 19.90%, respectively, *p* < 0.001) and circumferential (median value: −17.85, −15.09, and −13.66%, respectively, *p* < 0.001) directions (Figure 6). Longitudinal strain was similar between infarcted and adjacent region (−11.66 vs. −11.29%, *p* > 0.05) but both significantly worse than remote region (−16.45%; both *p* < 0.001).

### Intra-observer and Inter-observer Variability

Reproducibility was excellent for all strain parameters given in Table 3. The intraclass correlation coefficients ranged from 0.859 to 0.979 for intra-observer agreement and ranged from 0.749 to 0.954 for inter-observer agreement.

**Table 3:** Inter- and intra-observer variability of CMR feature tracking derived global and regional strain parameters.

	**Intra-observer**		**Inter-observer**	
	**ICC**	**95% CI**	**ICC**	**95% CI**
**RS, %**				
Global	0.916	0.773 to 0.971	0.9	0.736 to 0.965
Basal	0.859	0.578 to 0.953	0.749	0.416 to 0.907
Mid	0.924	0.788 to 0.974	0.883	0.693 to 0.959
Apical	0.869	0.651 to 0.954	0.886	0.690 to 0.960
**CS, %**				
Global	0.913	0.764 to 0.970	0.856	0.607 to 0.950
Basal	0.905	0.741 to 0.967	0.885	0.692 to 0.960
Mid	0.929	0.808 to 0.975	0.884	0.612 to 0.963
Apical	0.902	0.732 to 0.966	0.834	0.577 to 0.941
**LS, %**				
Global	0.924	0.716 to 0.976	0.913	0.679 to 0.973
Basal	0.979	0.921 to 0.993	0.954	0.871 to 0.984
Mid	0.9	0.736 to 0.965	0.886	0.637 to 0.963
Apical	0.92	0.768 to 0.973	0.9	0.732 to 0.965

*ICC, intraclass correlation coefficient; RS, radial strain; CS, circumferential strain; LS, longitudinal strain*.

## Discussion

The present study investigated the additional value and correlation of myocardial strain assessed by CMR feature-tracking to conventional CMR parameters in patients with a first anterior STEMI at 1 month after index procedure. The main findings were as follows: (1) compared with healthy controls, CMR feature-tracking detected impaired global strains in STEMI patients with normal LVEF; (2) strain was closely associated with infarcted myocardium detected by LGE, and global and regional strain could stratify different extent and transmurality of myocardial infarction respectively; (3) although without LGE, adjacent region had impaired strains comparing with remote region—deformation reduced successively from remote region to adjacent region and infarcted region.

Recently, myocardial strain analysis is considered as a powerful tool to quantify subtle and regional myocardial dysfunction over ejection fraction (6, 12). Speckle tracking echocardiography is a convenient way for strain analysis but is limited by inherent weakness of echocardiography, including angle-dependent, low signal/noise ratio and inter-vendor differences (13, 14). CMR tagging, requiring specific sequence, is time consuming and confined for clinical application. CMR feature-tracking analysis is a rapid and semi-automated approach performed offline on routine cine images, which has been popularly applied to research in cardiovascular diseases. Moreover, the feasibility of CMR feature-tracking and agreements with speckle tracking echocardiography, tagging, and strain-encoded MRI have been confirmed in several studies (15–18). However, better reproducibility of CMR feature-tracking was observed in global strain than segmental strain (19, 20). Also, a few studies showed a lower reproducibility of segmental strain by CMR feature-tracking than acquisition-based techniques including tagging and strain-encoded MRI (21, 22). Therefore, global deformation was more frequently applied in literatures. Fent et al. demonstrated that GLS was reduced in patients with previous myocardial infarction in the context of normal LVEF (4). However, they only conducted strain analysis on two-chamber and four-chamber cines to calculate longitudinal strain for 40 patients and 40 controls. Further, our study applied on three long-axis cines and short-axis cine stacks of LV demonstrated that GRS, GCS, and GLS were already declined in STEMI patients with normal LVEF, implying that these patients were supposed to receive active treatment to prevent further deterioration of cardiac function. Moreover, assessment of regional myocardial dysfunction is necessary and helpful for clinical strategy. Shah et al. demonstrated that about one-fifth of dysfunctional and thinned segments presented with negative LGE could recover from revascularization (23). In short, strain parameters are practicable and provide information about both global and regional myocardial dysfunction which is different from LVEF and may guide early therapy for better prognosis.

Previous studies demonstrated that strain parameters within 1 week post-STEMI showed moderate to strong correlation with LGE (*r* ranged from 0.32 to 0.64) and provided prediction of prognosis (24–26). However, edema and hemorrhage may result in overestimated infarct size by LGE soon after STEMI (27). The present study scheduled CMR examination 1 month after index event (the presence and extent of edema/hemorrhage were much less than acute period), and the results showed a strong association between strain parameters and LGE (GRS: *r* = 0.65; GCS: *r* = 0.69; GLS: *r* = 0.61). Moreover, the present study investigated the correlation of myocardial strain and LGE in detail. Our study showed that regional and global strain could discriminate different transmurality and extent of LGE, and segmental strains were good discriminators for LGE >50% (AUC: 0.763–0.903). A previous study reported that segments with >50% LGE extent were hard to recover despite successful revascularization (5). Therefore, it is an alternative to evaluate myocardial infarction without contrast administration and it is predominant for patients with contraindications to contrast agents. In addition, a few studies demonstrated that GLS provides incremental prognostic value to LGE (24, 25). Hence, strain parameters could provide useful information for clinical strategy.

Last but not the least, strain analysis could provide regional function of segments with different conditions. Götte et al. compared regional function between the infarcted and remote region and found significant differences by CMR tissue tagging but not by wall thickening analysis (11). An animal study, quantifying regional mechanical changes 2 weeks after index procedure by tagged cine, also presented similar results, in which circumferential strain was −1.5 ± 0.5%, −4.5 ± 0.8%, and −5.5 ± 1.4% in infarct zone, transition zone, and remote zone, respectively (direct comparison was not performed) (28). Our study performed in a larger human population and by a more convenient method (CMR feature-tracking) showed that strains in adjacent region were better than infarcted region and worse than remote region. The possible explanations were as follows: (1) under the background of function decline in infarcted region, kinetic coordination and synchrony of myocardium in adjacent region were impaired; (2) myocardial cells in adjacent region might be slightly edematous without LGE presence; (3) multivessel disease could have a chronic impact on the blood supply of uninvolved myocardium and impair the contractility of uninvolved myocardium. Also, strain impairment of ischemic segments has been demonstrated by a recent study, which investigated the discriminating ability of circumferential and longitudinal strain among ischemic, infarcted, and negative myocardium in patient and segmental levels (29). Furthermore, we speculated that considerably declined strain in adjacent region might contribute to negative remodeling of LV in long term, and the reduced deformation in adjacent region should be alerted.

### Study Limitations

There were several limitations in the present study. First, sample size was relatively small. Second, the study only focused on patients with first anterior STEMI so that it limited the ability to extrapolate the findings in a general acute coronary syndrome or STEMI population. Third, CMR parameters were retrospectively analyzed from a prospective cohort with LGE 1 month after percutaneous coronary intervention as study endpoint—T2/T2^*^ mapping was not scheduled so that edema and hemorrhage in the patients were not available; dynamic changes of CMR characteristics could not be observed; however, functional recovery, LV remodeling, and long-term functional outcome were valuable and could be assessed in follow-up CMR examinations (6 or 12 months). Moreover, prognostic results were not reported in the present study since the event rate was low. Therefore, the prognostic association between strain and LGE could not be investigated. Further prospective study with large numbers of patients might be warranted.

## Conclusion

Global and regional strain could stratify different extent and transmurality of LGE, respectively. Although without LGE, adjacent region had impaired strains comparing with remote region.

## Data Availability Statement

The original contributions presented in the study are included in the article/supplementary material, further inquiries can be directed to the corresponding author/s.

## Ethics Statement

The studies involving human participants were reviewed and approved by The Review Board of Fuwai Hospital. The patients/participants provided their written informed consent to participate in this study.

## Author Contributions

SY conception and design of study, analyzed images, and drafted the article. JZ conception and design of study, acquired clinical data, and drafted the article. KY, YZ, JS, and KJ analyzed images and critically revised the article. XC, HY, and SZ conception and design of study and critically reviewed the article. KZ performed statistical analysis and critically reviewed the article. PZ acquired clinical data and critically reviewed the article. All authors read and approved the final article for publication.

## Conflict of Interest

The authors declare that the research was conducted in the absence of any commercial or financial relationships that could be construed as a potential conflict of interest.

## Supplementary Material

Supplementary Figure 1 Comparison of global strain. ^*^*p* < 0.05 for comparison between two groups.
