# Supplementary figures and images for: Correlation of Myocardial Strain and Late Gadolinium Enhancement by Cardiac Magnetic Resonance After a First Anterior ST-Segment Elevation Myocardial Infarction
Source: Front Cardiovasc Med. 2021 Jul 2;8:705487. doi: 10.3389/fcvm.2021.705487 (PMC8282997; doi:10.3389/fcvm.2021.705487)

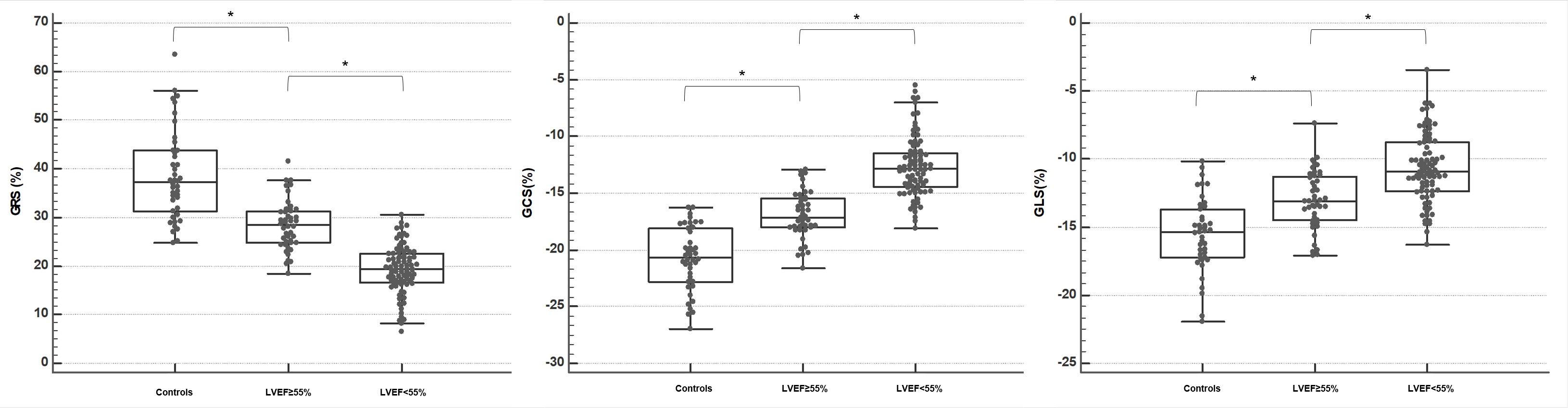

Supplement: Supplementary Figure 1 — Comparison of global strain. *p < 0.05 for comparison between two groups. [file Image_1.TIF]
